# Efficiency of the health extension programme in Tigray, Ethiopia: a data envelopment analysis

**DOI:** 10.1186/1472-698X-10-16

**Published:** 2010-06-14

**Authors:** Miguel San Sebastian, Hailemariam Lemma

**Affiliations:** 1 Umeå International School of Public Health, Department of Public Health and Clinical Medicine, Umeå university, SE-901 85 Umeå, Sweden; 2 Malaria Control Department, Tigray Health Bureau, P.O.Box 89, Mekelle, Tigray, Ethiopia

## Abstract

**Background:**

Since 2004, the government of Ethiopia has made a bold decision to strengthen and expand its primary health care system by launching the Health Extension Program (HEP). While the scaling up of the HEP is necessary to achieve the aim of universal access to primary health care, close attention should be paid to the performance of the program. Using a data envelopment analysis this study aimed at (i) to estimate the technical efficiency of a sample of health posts in rural Tigray, ii) to identify those factors which might be explaining the efficiency results.

**Methods:**

Efficiency was measured using a data envelopment analysis model. A Tobit model was performed to identify factors associated with efficiency. Seven rural districts (out of 35) were purposely chosen. Input/output information was collected from the database of the Tigray Health Bureau during July 2007-June 2008. Information was also collected on environmental factors that might influence the efficiency outcomes through a structured questionnaire from the correspondent district health officers.

**Results:**

Analysis was based on data from 60 health posts. The mean scores for technical and scale efficiency were 0.57 (SD = 0.32) and 0.95 (SD = 0.11) respectively. Out of the 60 health posts, 15 (25.0%) were found to be technically efficient constituting the best practice frontier. Thirty eight (63.3%) were operating at their most productive scale size. In the regression analysis, none of the variables was significantly associated with the efficiency outcome.

**Conclusion:**

There is a need to review the management of the health information system in the region. The findings have also revealed that only a quarter of the health posts are working efficiently and pointed the need for improvement. A closer monitoring of the health extension programme is required in order to achieve the best possible performance.

## Background

The last World Health Report (2008) has made a strong call to all the countries to direct their health systems towards the values of primary health care reflected in the Alma Ata declaration 30 years ago: social justice and the right to better health for all, participation and solidarity. Primary health care is considered to offer a comprehensive framework about how health systems can become more equitable, inclusive and fair [1]. To achieve these goals, good performing health systems regarding quality, access and efficiency are required.

Being the principal objective of a health system to improve people's health, one of the chief functions the system needs to perform is to deliver health services. It is therefore considered a major failing of the system when due to lack of efficiency, health interventions do not reach the populations that would benefit from them [2].

While the problem of scarcity of resources in the health systems, both human and financial, is a well acknowledged problem in the Africa region, there is a growing recognition that inefficiencies within the health care system abound [3]. Solving this lack of efficiency on the delivery of health services is not only an issue related to the sustainability of the health care system, but an immense responsibility of decision-makers to ensure that health delivery works best with the available resources.

Ethiopia is a federal state decentralized to regions. The main role of the Federal Ministry of Health is the development of policy including guidelines; resource mobilization, capacity development activities and technical support to regions. Regional Health Bureaus are responsible not only to adapt policies and guidelines to local situation, but to plan, monitor and evaluate the performance of the health system, to conduct operational research and to provide technical assistance to the districts. The districts are mainly the implementers of the plans. The health system in the country consists of a four-tier system; the Primary Health Care Unit (PHCU), the district hospital, the zonal hospital and the regional referral hospital. A PHCU is the grass-root level, which give preventive and curative health services for an average of 25 000 people. It includes a health centre and five satellite health posts [4].

Since 2004, the government of Ethiopia has made a bold decision to strengthen and expand its PHC system by launching the Health Extension Program (HEP). The HEP is designed to achieve significant basic health care coverage in the country over five years through the provision of a staffed health post to serve approximately 5 000 people. The aim of this new community-based health care delivery system is to improve access and equity in health care through a focus on sustained preventive health actions and increased health awareness. Every health post is staffed by two females Health Extension Workers (HEWs), who are high school graduated with an extra of one year training course. The training program for the HEWs includes 16 major packages under five components: (i) hygiene and environmental sanitation (i.e. construction, usage and maintenance of sanitary latrine), (ii) family health service (i.e. family planning, vaccination), (iii) disease prevention and control (i.e. HIV, TB, malaria), (iv) health education and communication and (v) nutrition. At this level, antipyretics, oral dehydration salts and antihelminthic and antimalarial drugs are available. Voluntary health workers (VHWs) (community health workers and traditional birth attendants) support HEWs in the health education activities of the different components in the community [5]. By May 2008, there were 24,500 HEWs trained and deployed which is 82% of the 30,000 target of the Ministry of Health in 2010/11 [6].

While the scaling up of the HEP is a necessary step to achieve the aim of universal access to primary health care, close attention should be paid to the performance of the program. Using a data envelopment analysis this study aimed at (i) to estimate the technical efficiency of a sample of health posts in the region of Tigray, ii) to identify those factors which might be explaining the efficiency results.

## Methods

### Study area

Tigray is the most northern regional state of Ethiopia bordering Eritrea and Sudan. The region covers 54,572 square kilometers ranging from low-arid to high-land (above 2200 masl) areas. Tigray is divided into 47 districts (woredas) which are grouped into six zones. Tigray has approximately 4.3 million inhabitants, most of them (81.2%) living in rural areas. The dominating religion in the region is Orthodox Christian (95.5%). In Tigray, 95% of the population is Tigrayan and their language, Tigrigna, is the working language in the region. The majority of the population works with agriculture. Famine and drought regularly strikes the region [7].

The health system in Tigray is essentially the same as in the rest of Ethiopia. There are five zonal hospitals, six district hospitals and one referral hospital in Mekelle, the capital. Malaria is the number one diagnosis for outpatient cases, followed by malnutrition and diarrheal and acute respiratory infections. The Regional Health Bureau initiated in 2005 an ambitious plan to achieve the universal coverage of the HEP. Currently, there are 1,220 health extension workers in Tigray and the coverage has increased from 30% in 2006/7 to above 70% in 2007/8 [8].

### DEA conceptual framework

To measure efficiency in health care organizations, two different frontier estimation methodologies, stochastic frontier analysis (SFA) and data envelopment analysis (DEA) are widely used in high income countries [9]. While there is no clear consensus to the best method to measure health facilities efficiency, several studies from low-income countries have pointed out the better suitability of the DEA for contexts where there is insufficient health sector information and particularly when the economic data is missing [10,11].

DEA is a non-parametric linear programming methodology for evaluating relative efficiency of each production unit among a set of fairly homogeneous decision-making units, e.g., district hospitals, health centres, etc. It sketches a production possibilities frontier (data envelop or efficient frontier) using combinations of inputs and outputs from best performing health facilities. Health facilities that compose the "best practice frontier" are assigned an efficiency score of one (or 100%) and are considered technically efficient compared to their peers. The efficiency of the health facilities below the efficiency frontier is measured in terms of their distance from the frontier. The inefficient health facilities are assigned a score between one and zero. The larger the score the more efficient a health facility is [12].

Osei et al (2005) have summarized the main strengthens and weakness of the methodology. The former includes: (i) it can handle multiple input and multiple output models/scenarios typical of hospitals and health centres; (ii) it does not require an assumption of a functional form relating inputs to output (as regression methods do); (iii) health facilities are directly compared against a peer or combination of peers; (iv) inputs and outputs can be very different units; (v) it does not require information on prices of inputs and outputs. The DEA has also some drawbacks: (i) it attributes any deviation from the "best practice frontier" to inefficiency, while some could be due to statistical noise, e.g. epidemics or measurement errors; (ii) given that DEA is a nonparametric technique, it is difficult to conduct statistical tests of hypotheses concerning the inefficiency and the structure of the production function [11,13].

### Model specification

Total efficiency is a combination of technical efficiency (TE), which reflects the ability of a unit to obtain maximal output from a set of inputs, and allocative efficiency, which reflect the ability of a unit to use the inputs in optimal proportions, given their respective prices. Due to the lack of economic information in this study, the calculation of the allocative efficiency was excluded. TE attempts to address two questions depending on whether it has input- or output-orientation. In input-oriented TE focuses on reducing input quantities used for a given level of outputs. On the other hand, in output-oriented TE the focus is on expanding output quantities with a fix amount of inputs. The choice of the approach is recommended to be based on which side of the orientation (input or outputs) the decision makers in the health facility have more control over [12]. In this study, we apply the output orientation model which addresses the question: by how much can the outputs be proportionally increased without changing the inputs quantities? The rational behind this selection was: i) the deployment of human resources (inputs) at the health post level is decided at national level without much influence capacity from the local level; ii) most of the chosen outputs are under control of the health post; HEWs have the mission to induce demand for preventive health care services (family planning, antenatal and delivery care) and promotion activities (education, visits by CHWs) and to create awareness about health services utilization at community and household level.

TE comprises both pure technical and scale efficiency components. Thus, a second consideration in estimating a DEA model is whether to assume constant or variable returns to scale (VRS). Under constant returns to scale (CRS), the interest is in productivity regardless of the scale of operations. VRS might be preferred if the interest is on the extent to which the scale of operations affects productivity or when not all units of analysis are considered to be operating at an optimal scale. Inappropriate size of a health facility (too large or too small) may sometimes be a cause for technical inefficiency. This is referred to as scale inefficiency and takes two forms - decreasing returns to scale (DRS) and increasing returns to scale (IRS). DRS implies that a health facility is too large for the volume of activities that it conducts. In order to operate at the most productive scale size, a health centre exhibiting DRS should scale down its scale of operation. In contrast, a health facility with IRS is too small for its scale of operation. If a health centre is exhibiting IRS, it should expand its scale of operation in order to become scale efficient. A health facility that is scale-efficient is said to operate under constant returns to scale [13,14]. Our analysis was carried out under the assumption of VRS.

### DEA variables

In the production process, health facilities turn inputs (factors of production) into outputs (health services). Inputs are usually divided into broad categories of labour (staff), materials (drugs) and capital (equipment). Despite there is a general consensus that the ultimate measure of output should be the change in the population health, practical difficulties (such as issues of measurement, access to data and health changes attributed to health care) make intermediate outputs a preferred option [11,15]. In our study the DEA model was estimated with a total of ten variables: two inputs and eight outputs. The two inputs were: (i) number of health extension workers and (ii) number of voluntary health workers (traditional birth attendants, community health workers). The eight outputs for each individual health post included: (i) number of health education sessions given by HEWs; ii) number of completed (three) antenatal care visits; (iii) number of child deliveries; (iv) number of persons that repeatedly visit the family planning service; (v) number of diarrheal cases treated in children under-five; vi) number of visits carried out by the community health workers; vii) number of total new patients attended and viii) number of malaria cases treated. The choice of inputs and outputs was guided by the components of the HEP.

### Data collection

The data used in this study are for the Ethiopian fiscal year 2000 (July 2007 - June 2008). Seven districts (out of 35 rural districts) were purposely chosen because of logistic reasons. These reasons were related to the quality of data available at the Tigray Health Bureau and the accessibility to the environmental variables at district level. One of the initial districts was rejected due to low quality data and substituted by another one. Input/output information was collected from the database of the Tigray Health Bureau.

Information was also collected on environmental factors that might influence the efficiency outcomes through a structured questionnaire from the correspondent district health officers. Personal characteristics of the HEWs such as marital status, pregnancy in the previous year, number of children and being born in the same subdistrict (tabia) were chosen to account for the individual variability of the health posts. Structural factors such as the population of the tabia, the distance to the health centre (in minutes walking) and the political support from the tabia's chief (little, moderate, high) were considered to account for population demographics, accessibility and political commitment respectively.

## Analysis

The technical efficiency scores were computed using the open-access Data Envelopment Analysis programme, version 2.1 (DEAP 2.1) designed by Coelli [16]. In order to use the less possible number of variables, a correlation analysis was conducted among the output variables. With a Spearman correlation coefficient greater than 0.6, none of the output variables was associated. To study whether there were extreme outliers which could affect the efficiency scores, we ran DEA analyses that dropped out each efficient health post one at a time from the analysis. The similarity of the efficiency ranking between the model prior to deleting any efficient health posts and the new models, was then tested by using the Spearman rank correlation coefficients. If the health posts are influential, a value of 0 would be obtained indicating no correlation. A value of 1 (or -1) would imply no influence of outliers on health post efficiency. This is a procedure called jackknifing and it tests the robustness of the DEA results in regard to outlier health posts [15,17]. To identify factors associated with the efficiency, the estimated technical efficiency scores obtained from the DEA was considered the dependent variable and regressed against the set of environmental variables using a Tobit model [13,18]. Coefficients with positive signs would indicate a positive correlation with efficiency. One might expect that being born in the tabia, being single, not pregnant last year and not having children would be associated with efficiency. Regarding structural factors, more population, less distance and more political commitment would also be positively correlated. This analysis was performed with Stata 10.0.

## Results

A total of 87 health posts belonging to seven districts fulfilled the requirements to be included in the sample. Health posts not operating in the previous year were excluded. Information from 27 (31.0%) health posts was incomplete or inconsistent, and thus analysis was based on data from 60 health posts. Summary statistics of the variables of interest are presented in Table 1. The results of the jackknifing analysis yield the value ranges of Spearman rank correlation coefficient from 0.90 to 1, which were significantly different from zero. This suggested that no efficient health post influenced the efficiency of the other health posts.

**Table 1:** Descriptive statistic of input and output variables, Tigray (July 2007 - June 2008)

Variable	Definition	Mean	Std. deviation	Minimum	Maximum
Input					
X1	# health extension workers	1.9	0.22	1	3
X2	# voluntary health workers	13.2	4.6	4	19
Outputs					
Y1	# health education sessions	241.93	411.88	1	2169
Y2	# women with completed antenatal care visits	14.71	23.13	1	128
Y3	# deliveries	25	25.5829	1	116
Y4	# family planning service	155.96	258.58	1	1287
Y5	# diarrheal cases treated	69.48	75.52	1	528
Y6	# visits by CHWs	27.66	98.93	1	577
Y7	# total patients attended	847.86	1081.63	182	8235
Y8	# malaria cases treated	401.33	537.47	42	3624

**Figure 1:**
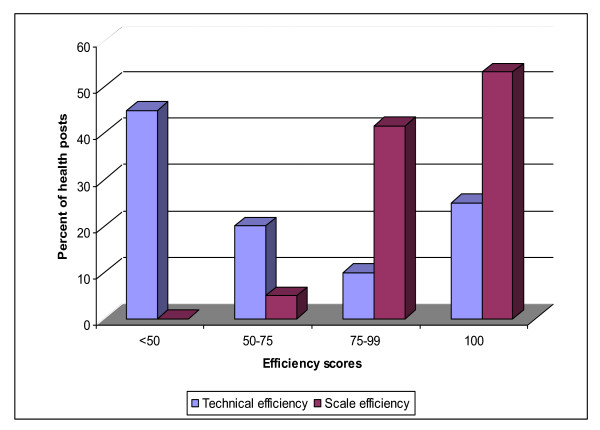
**Distribution of technical and scales efficiency scores of the health posts, Tigray (July 2007 - June 2008))**.

The VRS technical and scale efficiency scores for individual health posts are given in Table 2. Overall, the mean efficiency scores for TE and SE were 0.57 (SD = 0.32) and 0.95 (SD = 0.11) respectively. Out of the 60 health posts, 15 (25.0%) were found to be technically efficient constituting the best practice frontier. Twenty seven (60%) of the inefficient health posts had a TE score of less than 50% (Figure 1). The average TE score for the 45 inefficient health posts was 42% (SD = 0.23) meaning that these health facilities could potentially produce 58% more outputs using their current input endowment if they were to operate efficiently.

**Table 2:** Technical and scale efficiency scores and returns to scale characteristics of each health post, Tigray (July 2007 - June 2008)

Health post	Technical efficiency score	Scale efficiency score	Type of scale inefficiency
11403	1.000	1.000	-
20402	1.000	1.000	-
28401	1.000	1.000	-
28403	1.000	1.000	-
28404	1.000	1.000	-
43409	1.000	1.000	-
36402	1.000	1.000	-
36406	1.000	1.000	-
36407	1.000	1.000	-
36404	1.000	1.000	-
23411	1.000	1.000	-
11407	1.000	0.992	irs
36403	1.000	0.771	irs
20406	1.000	0.597	irs
35401	1.000	0.516	irs
43402	0.953	1.000	-
23409	0.912	1.000	-
28407	0.878	1.000	-
23422	0.818	1.000	-
23415	0.799	0.583	irs
23414	0.762	0.996	irs
36401	0.723	0.780	irs
23412	0.703	1.000	-
23410	0.651	1.000	-
20401	0.645	0.952	drs
28406	0.588	1.000	-
23416	0.587	1.000	-
43408	0.577	1.000	-
43405	0.559	0.999	-
23405	0.549	1.000	-
20404	0.544	0.983	drs
20403	0.513	0.965	drs
23401	0.509	0.998	drs
23421	0.422	1.000	-
23403	0.387	1.000	-
28412	0.384	0.999	-
28413	0.374	0.999	drs
20410	0.360	0.992	drs
23408	0.335	1.000	-
20409	0.312	0.936	drs
28402	0.307	0.996	drs
36405	0.295	0.960	drs
35407	0.291	0.839	irs
20405	0.286	0.888	drs
23406	0.277	1.000	-
23418	0.272	1.000	-
28411	0.269	0.999	-
28414	0.258	0.999	-
23417	0.257	0.999	-
35405	0.253	0.839	irs
28405	0.228	1.000	-
20407	0.227	0.910	drs
28408	0.217	1.000	-
23404	0.194	0.999	-
23407	0.167	1.000	-
28409	0.166	1.000	-
28410	0.146	1.000	-
23413	0.141	0.989	irs
35406	0.096	0.781	irs
35402	0.045	1.000	-

Of the total health posts in the data set, 38 (63.3%) exhibited constant returns to scale. In other words, they were operating at their most productive scale size. The rest 22 were considered as scale inefficient though most of them were close to the optimal size (85.0% had a score higher than 0.90). The scale inefficient health posts had an average SE score of 90% (SD = 0.14) implying that if these health posts had an optimal size, output would have increased by about 10% without increasing the input consumption. Eleven (18.3%) health posts manifested increasing returns to scale and another 11 (18.3%) decreasing returns to scale.

The results of the Tobit model for examining determinants of health posts' efficiency are given in Table 3. The individual variables being born in the tabia, marital status and pregnancy status were negatively associated with efficiency. Against our expectations, this means that a better performance was found in health posts where the HEWs were not locally born, both were married and had been pregnant last year. Lower efficiency scores corresponded to high number of children, less population, little support from the tabias'chief and less distance to the health post. Reasons why a greater distance from the health post is associated with a greater efficiency are not clear. None of the variables, however, was found to affect significantly the technical efficiency of the posts.

**Table 3:** Tobit regression analysis of potential factors influencing the technical efficiency scores

TE score	n (%)	Coef	Std. Err	p	95% CI
HEW from tabia					
None	27 (45.0)	Ref			
One	30 (50.0)	-0.063	0.116	0.588	-0.297 - 0.170
Both	3 (5.0)	-0.098	0.262	0.708	-0.626 - 0.428
HEW married					
Both	27 (45.0)	Ref			
One	21 (35.0)	-0.070	0.141	0.618	-0.354 - 0.212
None	12 (20.0)	-0.012	0.182	0.946	-0.380 - 0.355
HEW pregnant last year					
Both	6 (10.0)	Ref			
One	21 (35.0)	-0.295	0.198	0.143	-0.694 - 0.103
None	33 (55.0)	-0.139	0.210	0.510	-0.561 - 0.283
Number of children					
3-5	13 (21.6)	Ref			
1-2	33 (55.0)	0.086	0.131	0.512	-0.178 - 0.351
None	14 (23.3)	0.079	0.160	0.623	-0.243 - 0.402
Support from tabias' chief					
Little	16 (26.6)	Ref			
Moderate	27 (45.0)	0.044	0.130	0.733	-0.217 - 0.307
High	17 (28.3)	0.074	0.148	0.618	-0.223 - 0.372
Tabia population*	5850.6 (1469.8)	0.000	0.000	0.487	-0.000 - 0.000
Distance to health post*	11.9 (7.2)	0.010	0.007	0.186	-0.005 - 0.026
_cons		0.480	0.331	0.154	-0.186 - 1.147
/sigma		0.376	0.042		0.291 - 0.462

* mean (standard deviation)

## Discussion

The first main finding of this study is related to the lack of quality of the health information available at the central level of Tigray Health Bureau. The information of all the health posts of one district and 27 health posts of the other seven districts could not be used. Reasons need to be further explored but they could be attributed to inadequate data collection and reporting from the HEWs or inadequate data entry at the central level.

The second main result is the poor performance of this sample of health posts with most of them (75.0%) carrying out activities at less than optimal levels. Health posts could therefore increase on the number of preventive and curative activities with the resources they currently have. Though previous research in Tigray has shown a certain satisfaction of the community with the health services provision by the HEWs [19], the study was focused in only one district not covered in our sample. Most of the health posts were however operating to their most productive scale sizes (CRS) for the outputs produced which suggest that the human resources allocated to the health posts (2 HEWs and around 13 VHWs) might be enough at this stage.

In recent years, an increase number of studies have been conducted measuring efficiency using DEA in Africa, few however at the primary health care level. In Kenya, a study found 56% health centres to be technically inefficient and 70% scale inefficient [20]. Seventy per cent of primary health care clinics in rural South Africa were found to be technically inefficient and 84% scale inefficient [21]. Another study in Sierra Leone reported that 59% of the 37 peripheral health units were technically inefficient [22]. Two studies from Ghana and Seychelles have recently been published; the former showed that 65% of the health centres were technically and 79% scale inefficient while the latter found that 41% and 59% of the health centres were run inadequately for technical and scale efficiencies [14,23]. While data between countries are not comparable because the selection of input/output variable is context-based and the socio-economic conditions of the different countries vary enormously, the low scores observed in our study raises concerns about the efficiency of the HEP in the region.

The regression analysis did not identify potential factors associated with the efficiency of the health posts. A study conducted at the initial stages of the HEP in the country pointed out some of the reasons that could be explaining our findings. The challenges mentioned included the harmonization of the staff patterning at the health post level, and the lack of clear guidelines on relationship with other workers at the community level, on career structure, transfer and leave of absences. The reporting and health management information system was also a great weakness [24]. Further research will be required in order to confirm if these issues are relevant for Tigray.

### Limitations of the study

Several limitations should be noted. First, output data were collected in order to represent the broad range of activities of the HEWs; however, the selection of other outputs might have produced different efficiency outcomes. Second, the information on input prices could not be collected and thus, the allocative efficiency not estimated. Third, inputs and outputs were gathered for only one period which made not possible to observe any impact of the HEP. Four, given that the study was conducted in a sample of districts the generalization of the results to the Tigray region should be taken cautiously. Two final limitations are related to the regression method used. Since there is no generally accepted method for taking into account environmental variable in DEA models, a Tobit regression model was adopted; we are aware that concerns regarding its applicability have been raised [13]. The relative small size could have hidden some statistical significant relationship between the environmental factors and the efficiency scores.

## Conclusion

This study has shown the need to review the management of the health information system in the region. The findings have also revealed that only 25.0% of the health posts are working efficiently and have indicated those health posts that need improvement. The potential for a better performance of the different components of the HEP is of great public heath importance given the scarce resources and poor health indicators of the region. Discussions have already initiated within the Tigray Health Bureau in order to improve the situation. In line with other research, DEA has shown to be an easy, affordable and accessible tool to assess the performance of health systems. The government of Ethiopia is making an enormous effort to extend affordable primary health care services across the country. This achievement however should not be done in detriment of its efficiency. Whether these results apply regionally or nationally should be explored and the performance of the HEP carefully monitored.

## Competing interests

The authors declare that they have no competing interests.

## Authors' contributions

MSS developed the idea, analysed the data and drafted sections of the manuscript. HL collected the data and participated in drafting sections of the manuscript. All the authors read and approved the final manuscript.

## Pre-publication history

The pre-publication history for this paper can be accessed here:

http://www.biomedcentral.com/1472-698X/10/16/prepub
